# Complete Enzymatic Depolymerization of Polyethylene Terephthalate (PET) Plastic Using a *Saccharomyces cerevisiae-*Based Whole-Cell Biocatalyst

**DOI:** 10.1021/acs.estlett.5c00190

**Published:** 2025-03-19

**Authors:** Siddhant Gulati, Qing Sun

**Affiliations:** † Department of Chemical Engineering, Texas A&M University, College Station, Texas 77843, United States; ‡ Interdisciplinary Graduate Program in Genetics and Genomics, Texas A&M University, College Station, Texas 77843, United States

**Keywords:** cellulosome, enzymatic degradation, polyethylene terephthalate (PET), whole-cell biocatalyst, yeast surface display, complete depolymerization

## Abstract

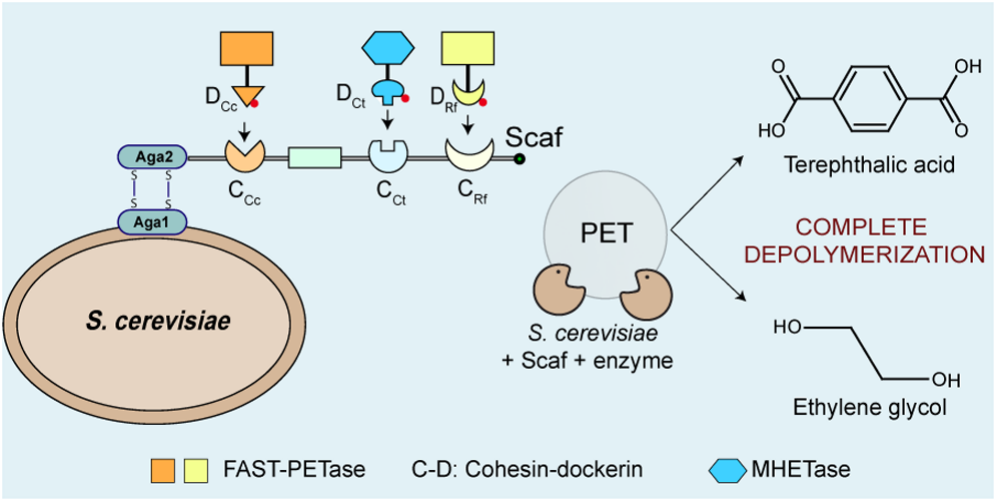

Management of polyethylene terephthalate (PET) plastic waste remains a challenge. PET-hydrolyzing enzymes (PHEs) such as *Is*PETase and variants like FAST-PETase demonstrate promising PET depolymerization capabilities at ambient temperatures and can be utilized to recycle and upcycle plastic waste. Whole-cell biocatalysts displaying PHEs on their surface offer high efficiency, reusability, and stability for PET depolymerization. However, their efficacy in fully breaking down PET is hindered by the necessity of two enzymes: PETase and MHETase. Current whole-cell systems either display only one enzyme or struggle with performance when displaying larger enzymes such as the MHETase–PETase chimera. We developed a *Saccharomyces cerevisiae*-based whole-cell biocatalyst for complete depolymerization of PET into its constituent monomers with no accumulation of intermediate products. Leveraging a cellulosome-inspired trifunctional protein scaffoldin displayed on the yeast surface, we co-immobilized FAST-PETase and MHETase, forming a multi-enzyme cluster. This whole-cell biocatalyst achieved complete PET depolymerization at 30 °C, yielding 4.95 mM terephthalic acid (TPA) when tested on a PET film. Furthermore, we showed improved PET depolymerization ability by binding FAST-PETase at multiple sites on the scaffoldin. The whole cells had the added advantage of retained activity over multiple reusability cycles. This breakthrough in complete PET depolymerization marks a step toward a circular plastic economy.

## Introduction

Polyethylene terephthalate (PET) plastic has become ubiquitous in daily life, with an estimated annual global production of 82 million tonnes.^1^ However, since only a small fraction of this produced plastic is recycled (<20%), PET waste management is a daunting challenge.^2^ PET can be recycled via mechanical, chemical, or biological methods.^3,4^ Enzymatic biocatalysis is emerging as a green route for plastic waste recycling and could also open up new avenues for “upcycling” waste into higher-value products.^5,6^

Many PET-hydrolyzing enzymes (PHEs) have been identified and engineered for enzymatic PET recycling.^7,8^ The mesophilic enzyme *Is*PETase, secreted by *Ideonella sakaiensis*, exhibited PET depolymerization at ambient temperatures.^9^ Engineered *Is*PETase variants with improved activity and thermostability have since been developed,^10,11^ with FAST-PETase^12^ exhibiting superior performance. Whole-cell biocatalysis is a promising approach to achieving PET depolymerization using enzymatic systems. By displaying PET-degrading enzymes on the surface of a prokaryotic or eukaryotic cell, promising PET depolymerization performance has been obtained.^13−18^ The benefits of these systems include improved activity due to reduced enzyme aggregation,^14^ reusability, and enhanced enzyme stability.^19^ Aggregation of free PETase at high concentrations (>100 nM) significantly limits its performance, and enzyme immobilization is an effective strategy to reduce aggregation and improve PET degradation activity.^9,14^

However, to achieve complete depolymerization of PET into its constituent monomers, terephthalic acid (TPA) and ethylene glycol (EG), without the accumulation of intermediate products, enzymatic partner MHETase is also required.^9^ PETase and MHETase work synergistically to completely degrade PET, with MHETase–PETase fusion enzymes exhibiting even better performance than free enzyme mixtures.^20,21^ Additionally, the accumulation of intermediate product mono(2-hydroxyethyl) terephthalate (MHET) in the absence of MHETase inhibits enzyme activity.^21−23^ A major drawback of existing PETase-based whole-cell biocatalysts is their ability to display only one enzyme efficiently on their surface.^13−17^ Poor performance^18^ or incomplete conversion^24^ is observed when larger passenger enzymes like the MHETase–PETase chimera are displayed on the surface. Thus, complete PET depolymerization into its constituent monomers with no accumulation of intermediates remains challenging in such systems.

To efficiently hydrolyze recalcitrant polymers like cellulose, certain anaerobic bacteria have developed multienzyme complexes called cellulosomes.^25^ Inspired by these natural systems, artificial, designer cellulosomes have been developed by recruiting necessary cellulose-hydrolyzing enzymes on a microbial surface scaffold with sequence, distance, and ratio specificity, exhibiting superior performance compared with free enzymes.^26−28^

In this work, we developed a cellulosome-inspired *Saccharomyces cerevisiae*-based whole-cell biocatalyst system capable of complete depolymerization of PET to TPA and EG. Previous research has demonstrated the excellent surface display capabilities of *S. cerevisiae*-based systems.^29,30^ Additionally, the scaffoldin-based approach eliminates the need for exhaustive experimental screening to surface display enzymes, thus rendering it highly direct.^28,31,32^ This approach also harnesses the benefits of enzyme proximity, substrate channeling, and synergy, which has led to efficient substrate degradation in cellulosome-based systems.^33,34^ FAST-PETase and MHETase were bound on a trifunctional protein scaffold displayed on the yeast cell surface through high-affinity cohesin–dockerin interactions. We confirmed the scaffoldin’s display on the yeast cell surface and successful binding of each enzyme to this scaffoldin. Subsequently, we assessed the PET depolymerization activity of this biocatalyst and showcased its capability to completely break down PET into TPA and EG. Finally, we evaluated the reusability of the whole-cell biocatalyst for enzymatic activity assays after separating it from the reaction medium via centrifugation. This work is valuable for achieving eco-friendly plastic waste management.

## Methods and Materials

### Yeast Culture and Display of Trifunctional Scaffoldin on the Yeast Cell Surface

*S. cerevisiae* strain EBY100 (*MAT*a *AGA1*::*GAL1-AGA1*::*URA3 ura3*-*52 trp1 le*u2-*1 his3*-*200 pep4*::*HIS3 prb1*-1.*6R can1 GAL*) transformed with pScaf-ctf^26^ was used for the surface display of the trifunctional scaffoldin. *S. cerevisiae* EBY100 without the pScaf-ctf plasmid served as a negative control. Detailed information regarding culture conditions is provided in Section S2 of the Supporting Information.

### Expression and Purification of PET-Degrading Enzymes

Sequences of gene fragments encoding dockerins (Doc) from *Clostridium cellulolyticum* (Doc Cc), *Clostridium thermocellum* (Doc Ct), and *Ruminococcus flavefaciens* (Doc Rf) were obtained using a process from a previous publication.^26^ Dockerin-tagged FAST-PETase and MHETase were constructed by fusing the dockerins at the C-terminus of the protein of interest separated by a flexible GS linker and transformed into *Escherichia coli* NEB 5-alpha. The constructed plasmids were transformed into *E. coli* SHuffle T7 for protein expression. Detailed information regarding induction conditions is provided in Section S3.

After protein expression and cell lysis, the proteins were purified by His-tag purification, followed by concentration and buffer exchange into exchange buffer (50 mM HEPES, 100 mM NaCl, pH 8). Protein purity was assessed using sodium dodecyl sulfate–polyacrylamide gel electrophoresis (SDS–PAGE) (Figure S4) and quantified using Bio-Rad Image Lab software. The protein concentration was determined using a Bio-Rad DC Protein Assay.

### Enzyme Assembly on the Yeast Cell Surface

Yeast cells displaying the scaffoldin were resuspended in binding buffer (50 mM Tris HCl, 100 mM NaCl, 10 mM CaCl_2_, pH 8). Subsequently, purified dockerin-tagged enzymes were added and incubated with the yeast cells at 20 °C for 1.5 h with continuous shaking. After incubation, the yeast cells were washed with 1× phosphate-buffered saline (PBS) to remove unbound dockerins and resuspended in binding buffer for activity assays.

### Enzyme Activity Assays on PET

Amorphous PET film (Goodfellow ES301445, crystallinity of 4.2 ± 2%, number-average molar mass (*M*_n_) of 19.7 ± 8.3 kDa, weight-average molar mass (*M*_w_) of 33.8 ± 6.8 kDa)^22^ was used as a substrate to test the PET hydrolytic activity. Circular PET films (diameter of 6 mm, initial weight of 9 mg) were washed with 1% SDS, 20% ethanol, and deionized water prior to use. One film was added to a glass test tube containing 300 μL of yeast cells (OD_600_ = 10) (Figure S3) and incubated at 30 °C and 250 rpm on an orbital shaker. Samples were collected periodically to quantify the reaction products.

To obtain samples for the analysis of PET degradation products, the reaction mixture was separated from the PET film and centrifuged to remove yeast cells. The supernatant thus obtained was heated at 85 °C for 20 min to ensure that there was no residual enzyme activity, followed by analysis using a HPLC instrument equipped with a diode array detector (detection wavelength of 240 nm). MHET and TPA formation was quantified by comparison against a standard curve prepared using commercially available standards. Detailed information regarding the HPLC method is provided in Section S3.

### Confirmation of the Scaffoldin Surface Display and Determination of the FAST-PETase Saturation Concentration

To confirm the surface display of the scaffoldin, *S. cerevisiae* cells (OD_600_ = 1) were centrifuged and washed with 1× PBS. The cells were resuspended in 100 μL of blocking buffer (1× PBS containing 1 mg/mL bovine serum albumin (BSA)) and left on a nutator for 1 h for blocking, followed by incubation with the primary anti-C-myc antibody (#2278, Cell Signaling Technology) at 4 °C overnight. The cells were then washed with 1× PBS and resuspended in blocking buffer, followed by incubation with an Alexa Fluor 488-conjugated secondary antibody (#4412, Cell Signaling Technology) at room temperature for 2 h in the dark. After washing with 1×PBS, the fluorescence was measured using a microplate reader (λ_ex_ = 488 nm; λ_em_ = 530 nm).

To determine the saturation concentration of FAST-PETase-Dockerin Rf (FP-Rf) and FAST-PETase-Dockerin Cc (FP-Cc) on the scaffoldin, equimolar amounts of both enzymes were bound to the scaffoldin using the enzyme assembly protocol. *S. cerevisiae* cells (OD_600_ = 5) containing the bound dockerins were centrifuged, washed, and then blocked for 1 h in blocking buffer. They were then incubated with the anti-C-His antibody conjugated to Alexa Fluor 488 (#14930, Cell Signaling Technology) for 1 h at room temperature in the dark. The fluorescence was measured after washing with 1×PBS (λ_ex_ = 488 nm; λ_em_ = 530 nm).

### Reusability of the Whole-Cell Biocatalyst

*p*-Nitrophenyl acetate (*p*NPA) was used as a substrate to determine the relative activity of new and reused whole cells. First, 20 μL portions of yeast cells (OD_600_ = 20) bound with all three enzymes were added to 175 μL of PBS, followed by addition of 5 μL of *p*NPA in DMSO (final *p*NPA concentration of 1 mM, 30 °C) in a 96-well plate. *p*-Nitrophenol (*p*NP) release was monitored by measuring the absorbance at 405 nm using a microplate reader. The enzyme activity was calculated as the ratio of the slope of the linear region of the *p*NP release curve and OD_600_ of the cells. After each *p*NPA hydrolysis cycle, the yeast cells were centrifuged and washed with 1×PBS to remove residual components from the previous cycle.

## Results

### Functional Display of Scaffoldin on the Surface of the Yeast Cell

**Figure 1:**
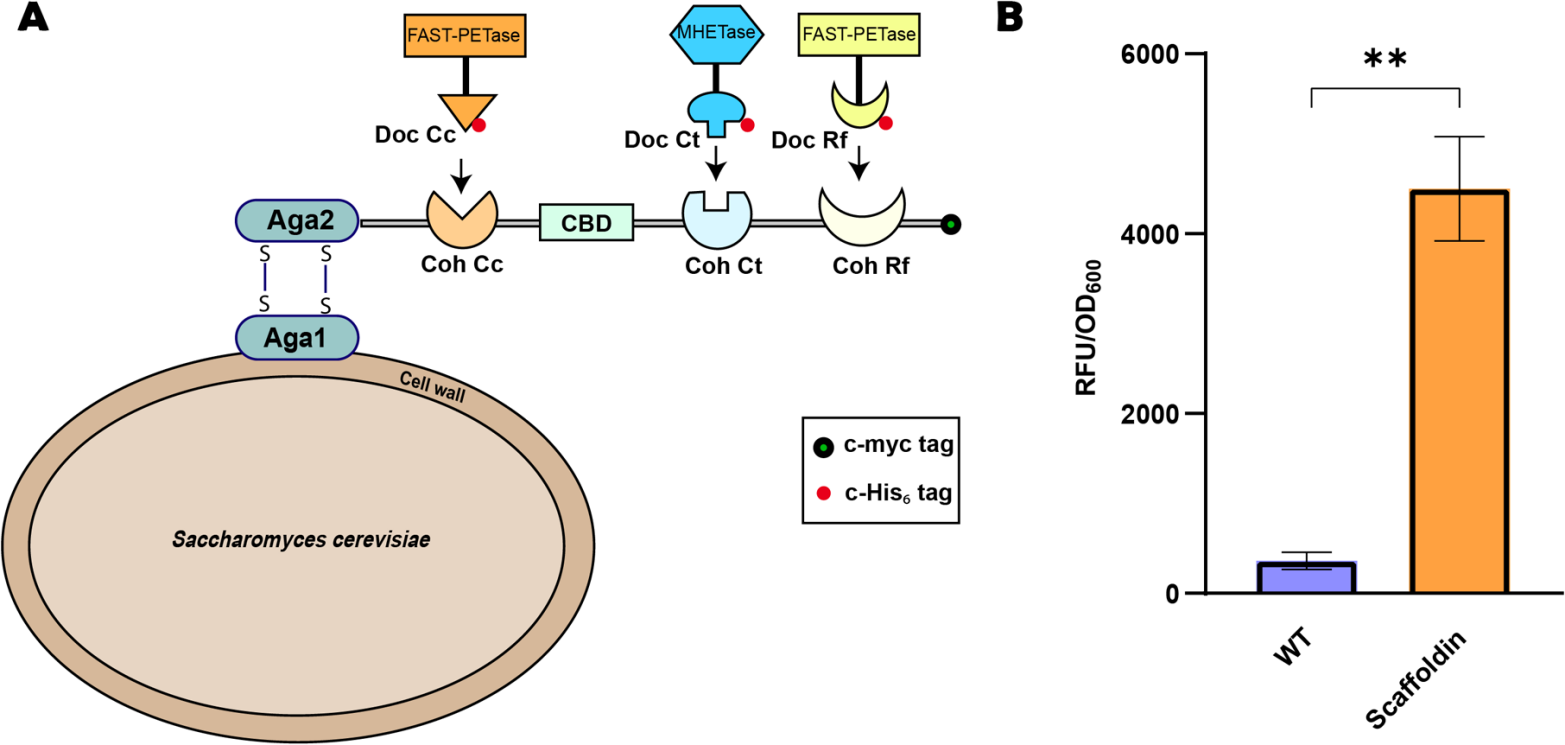
(A) Schematic representation of a trifunctional scaffoldin displayed on the yeast cell surface. The scaffoldin consists of cohesin domains derived from *C. cellulolyticum* (Coh Cc), *C. thermocellum* (Coh Ct), and *R. flavefaciens* (Coh Rf) and a cellulose-binding domain (CBD). Dockerin-tagged FAST-PETase and MHETase bind specifically to their corresponding cohesin domains. (B) Fluorescence intensity of scaffoldin-displaying whole cells probed using a primary anti-C-myc antibody and an Alexa Fluor 488-conjugated secondary antibody. Wild-type (WT) yeast cells served as the negative control. Experiments were conducted in triplicate, and data shown are mean values (±standard deviation). Statistical significance was evaluated using unpaired Student’s *t* tests. **P < 0.01.

A trifunctional scaffoldin consisting of three cohesin (Coh) domains from *C. cellulolyticum* (Coh Cc), *C. thermocellum* (Coh Ct), and *R. flavefaciens* (Coh Rf) with a cellulose-binding domain (CBD) from *C*. *thermocellum* was displayed on the surface of the *S. cerevisiae* cell using the Aga1–Aga2 system (Figure 1A).^26^ A C-myc tag on the scaffoldin was utilized to verify its surface display. Upon incubating *S. cerevisiae* with a fluorophore-conjugated anti-C-myc antibody, a considerably higher fluorescence intensity was observed in the scaffoldin-displaying cells compared to wild-type whole cells (Figure 1B), confirming the surface localization of the scaffoldin.

### Binding of PET-Degrading Enzymes on the Scaffoldin

Previous studies have reported that high PETase loading leads to high degradation performance and even low MHETase concentrations are sufficient to boost product release and convert all MHET into TPA.^20^ Based on this understanding, we hypothesized that attaching FAST-PETase at two different sites would allow higher FAST-PETase concentrations than single-site attachment of FAST-PETase, promoting efficient PET depolymerization. We utilized FP-Rf and FP-Cc to immobilize FAST-PETase on the surface scaffoldin and MHETase-Doc Ct (MH-Ct) for MHETase immobilization (Figure 1).

We determined the saturation concentration of FAST-PETase on the scaffoldin by incubating varying concentrations of FP-Rf and FP-Cc with yeast cells (Figure S1). This concentration of the FAST-PETase dockerin enzymes was used for activity assays on the PET film. Simultaneously, MH-Ct was co-incubated with these yeast cells.

### PET Depolymerization Performance of the Whole-Cell Biocatalyst

**Figure 2:**
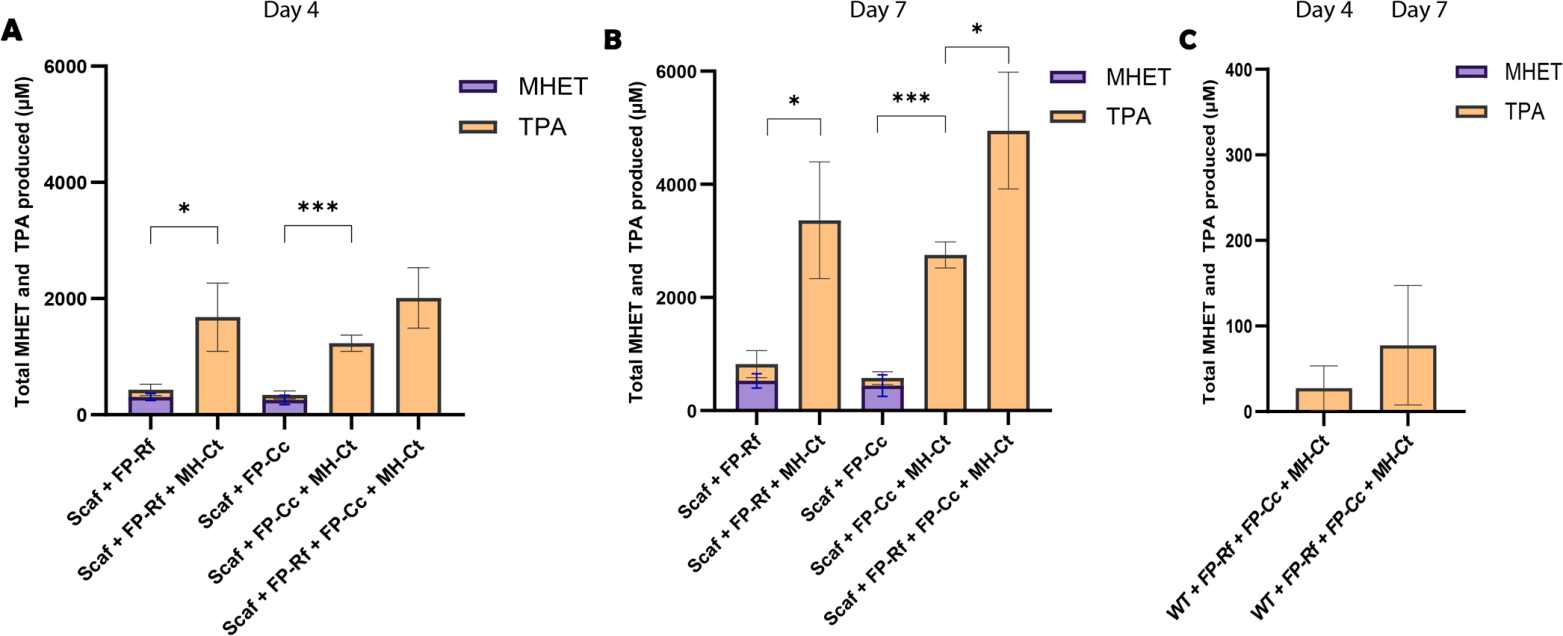
Determination of the PET hydrolytic activity of the whole-cell biocatalyst, evaluated by quantification of the amount of MHET and TPA released in the reaction supernatant via HPLC after degradation of the PET film by enzyme-bound scaffoldin-displaying cells on (A) day 4 and (B) day 7 and (C) WT yeast cells. Reactions were performed at 30 °C in binding buffer (whole-cell OD_600_ = 10, FP-Rf = 400 nM, FP-Cc = 400 nM, MH-Ct = 50 nM). Experiments were conducted in triplicate, and data shown are mean values (±standard deviation). Statistical significance was evaluated using unpaired Student’s *t* tests. *P < 0.05; ***P < 0.001.

Following the incubation of *S. cerevisiae* whole cells with the PET film substrate, we analyzed the supernatant using HPLC to monitor MHET and TPA formation. When only FAST-PETase dockerins were present on the scaffoldin, both MHET and TPA were formed (Figure 2, Figure S2). When MH-Ct was present on the scaffoldin along with FP-Cc or FP-Rf, we observed complete PET depolymerization, yielding only TPA as the reaction product with no accumulation of MHET or other reaction intermediates (Figure 2, Figure S2). Since MHETase, even in a small amount relative to FAST-PETase, is required for the degradation of PET to its constituent monomers, this demonstrates that each enzyme, including FAST-PETase and MHETase, was active after binding to the scaffoldin, and the PETase–MHETase pair was able to completely depolymerize PET to TPA and EG. As expected, MH-Ct alone did not show any activity on the PET film (data not shown).

Meanwhile, when wild-type yeast cells (without scaffoldin) were incubated with all three enzymes, we observed negligible TPA formation compared to the scaffoldin-displaying cells. This confirmed minimal nonspecific enzyme binding on the yeast surface in the absence of the scaffoldin (Figure 2C).

Total product formation was significantly boosted when MH-Ct was co-immobilized with FP-Rf or FP-Cc (Figure 2A,B). This could be attributed to the inhibitory effect of MHET accumulation in the absence of MHETase, which has been shown to detrimentally impact PETase activity.^21,22^

We also explored the effect of having two FAST-PETases and one MHETase on the scaffoldin to further enhance the concentration of the FAST-PETase in the depolymerization reaction. The three-enzyme system exhibited up to 1.6 times better depolymerization performance than the two-enzyme counterparts throughout day 4, with mean TPA yields of 2 mM. This improvement in product yields can be attributed to cooperative PET depolymerization by both FAST-PETase dockerins.

Throughout day 7 (Figure 2B), the whole cells remained active, with an up to 2.4-fold increase in TPA production relative to day 4. Notably, two FAST-PETases and one MHETase on the scaffoldin proved to be extremely beneficial, resulting in a 1.8-fold increase relative to that of FP-Cc (with MH-Ct). When all three enzymes were bound to the scaffoldin, a TPA yield of 4.95 mM was obtained after incubation with the PET film for 7 days. This corresponds to a degradation rate of 124.7 μg day^–1^ cm^–2^, which is 4.7-fold higher than the highest reported whole-cell biocatalyst degradation rate observed by an *E. coli*-based surface display system using the YeeJ autotransporter under its optimum conditions on an amorphous PET film.^14^ Thus, we successfully demonstrated a highly efficient yeast-based whole-cell biocatalyst system that could simultaneously immobilize multiple FAST-PETases and MHETase on the cell surface for efficient and complete depolymerization of PET to its monomeric constituents. The high surface display efficiency of yeast, ease of genetic modification and culturing, and generally regarded as safe (GRAS) status make it an extremely advantageous host for bioremediation.^35^

### Reusability of Whole-Cell Biocatalysts

**Figure 3:**
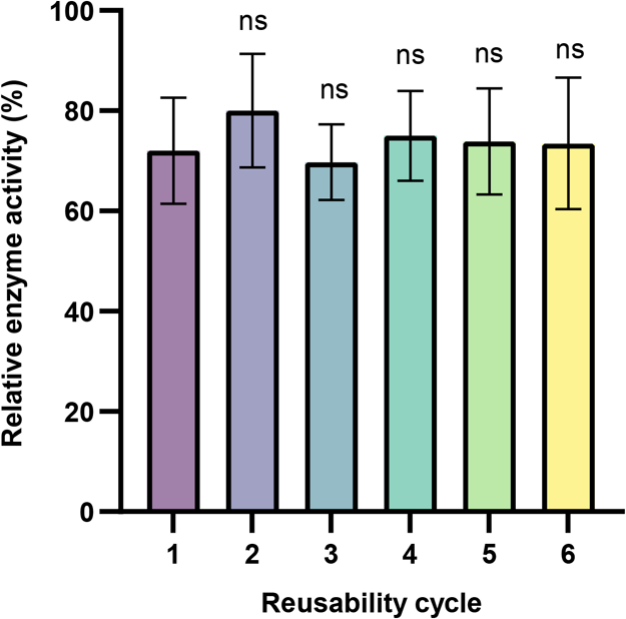
Reusability of the whole-cell biocatalyst using *p*NPA as a substrate. Experiments were conducted in triplicate, and data shown are mean values (±standard deviation). Enzyme activity from fresh, non-reused cells was taken as 100%. Statistical significance was evaluated using unpaired Student’s *t* tests. ns means not significant.

Whole-cell biocatalysts can be easily separated from the reaction medium through centrifugation, filtration, and sedimentation, facilitating use over multiple reaction cycles.^16^ The reusability of this yeast biocatalyst was determined by using a *p*NPA hydrolytic assay. The cells were recovered easily by centrifugation and showed promising reusability. Although we observed a nearly 25% decrease in activity in the first reusability cycle, the biocatalyst retained activity over five subsequent cycles (Figure 3). This strongly demonstrates that the whole-cell biocatalyst can be reused for multiple rounds.

## Discussion

Incomplete depolymerization with accumulation of reaction intermediates^24^ or compromised activity^18^ had proven to be a challenge for existing surface display systems being used for PET degradation. Our work leveraged the high surface display efficiency of *S. cerevisiae* and the benefits of enzyme proximity and substrate channeling provided by a protein scaffoldin for directed co-assembly of PET-degrading enzymes for efficient PET depolymerization. Co-immobilization of FAST-PETase and MHETase on a surface-displayed trifunctional scaffoldin led to complete depolymerization of PET into its constituent monomers TPA and EG, with no accumulation of reaction intermediates. In our system, the presence of MHETase along with FAST-PETase led to a nearly 5-fold boost in product formation, and attachment of FAST-PETase at two sites on the trifunctional scaffoldin further boosted yields by up to 1.8-fold. TPA yields of 4.95 mM were obtained in 7 days on an amorphous PET film with no pretreatment requirement. Whole-cell activity was retained over at least six reusability cycles.

Produced environmentally benign monomers TPA and EG can be reused by chemical and biological methods. They can be used to regenerate virgin PET, enabling closed-loop PET recycling.^12^ Given the high purity of the reaction supernatant after depolymerization, the constituent monomers can be potentially directly utilized without additional separation and purification steps. Furthermore, the whole-cell biocatalyst can also be easily separated by centrifugation on a small scale or by sedimentation, filtration, and decantation on a large scale.^16^ Monomers TPA and EG can be upcycled into value-added products like the biodegradable plastic polyhydroxyalkanoate (PHA)^36,37^ or intermediates like protocatechuic acid (PCA), gallic acid (GA),^38^ and lycopene,^2^ which serve as precursors for many industrially valuable products. This upcycling can be done using a single strain^2^ or a microbial community of biocatalysts.^37^ Alternatively, the whole-cell catalyst can be integrated with existing chemo-bioprocesses for one-pot PET upcycling.^39^ This biocatalyst system has the unique potential to be used as a biotransformation platform to recycle and upcycle PET waste, since the use of divergent cohesin–dockerin pairs can facilitate directed assembly of multiple enzyme cascades on the cell surface.^40^ The whole-cell biocatalyst can also be used to degrade microplastics in wastewater effluents.^16^ Thus, this PET depolymerization approach could pave the way for a circular plastic economy.

## Supporting Information Available

List of abbreviations used in the main text of the manuscript (Section S1), yeast culture conditions and growth media (Section S2), induction conditions for expression of PET-degrading enzymes (Section S3), HPLC method for analysis of enzymatic PET degradation products (Section S4), determination of the saturation concentration of FAST-PETase on the yeast scaffoldin (Figure S1), HPLC chromatograms of the TPA standard, MHET standard, and PET degradation products on day 4 (Figure S2), reaction setup for PET depolymerization (Figure S3), and SDS–PAGE, Western blot, and yield of PET-degrading enzymes after His-tag purification (Figure S4) (PDF)
