# Supplementary material for: Complete Enzymatic Depolymerization of Polyethylene Terephthalate (PET) Plastic Using a Saccharomyces cerevisiae-Based Whole-Cell Biocatalyst
Source: Environ Sci Technol Lett. 2025 Mar 19;12(4):419–24. doi: 10.1021/acs.estlett.5c00190 (PMC11984091; doi:10.1021/acs.estlett.5c00190)
Supplement: Supplementary file 1 — ez5c00190_si_001.pdf [file ez5c00190_si_001.pdf]

Supporting information

Complete enzymatic depolymerization of polyethylene terephthalate (PET) plastic using a *Saccharomyces cerevisiae*-based whole-cell biocatalyst

Siddhant Gulati[1], Qing Sun[1,2]*

[1]Department of Chemical Engineering, Texas A&M University, College Station TX 77843, United States

[2]Interdisciplinary Graduate Program in Genetics and Genomics, Texas A&M University, College Station, TX, 77843, United States

*Corresponding author - sunqing@tamu.edu

Table of contents:

Section S1: List of abbreviations used in the main text of the manuscript

Section S2: Yeast culture conditions and growth media

Section S3: Induction conditions for expression of PET-degrading enzymes

Section S4: HPLC method for analysis of enzymatic PET degradation products

Figure S1: Determination of saturation concentration of FAST-PETase on the yeast scaffoldin

Figure S2: HPLC chromatograms of TPA standard, MHET standard, and PET degradation products on Day 4

Figure S3: Reaction setup for PET depolymerization

Figure S4: SDS-PAGE, Western Blot and yield of PET-degrading enzymes after Histag purification

Section S1: List of abbreviations used in the main text of the manuscript

**BSA**	Bovine Serum Albumin
**CBD**	Cellulose-binding domain
**Cc**	*Clostridium cellulolyticum*
**Ct**	*Clostridium thermocellum*
**Coh**	Cohesin
**Doc**	Dockerin
E. coli	*Escherichia coli*
**EG**	Ethylene glycol
**FP-Cc**	FAST-PETase-Dockerin Cc
**FP-Rf**	FAST-PETase-Dockerin Rf
**GRAS**	Generally Regarded As Safe
**HPLC**	High-performance liquid chromatography
**MH-Ct**	MHETase-Dockerin Ct
**MHET**	mono(2-hydroxyethyl) terephthalate
**PBS**	Phosphate buffered saline
**PET**	Polyethylene terephthalate
**PHEs**	PET-hydrolyzing enzymes
***p*NP**	*p*-nitrophenol
***p*NPA**	*p*-nitrophenyl acetate
**Rf**	*Ruminococcus flavefaciens*
S. cerevisiae	*Saccharomyces cerevisiae*
**SDS-PAGE**	Sodium dodecyl sulfate-polyacrylamide gel electrophoresis
**TPA**	Terephthalic acid
**WT**	Wild-type

Section S2: Yeast culture conditions and growth media

S. cerevisiae EBY100 cells harboring the surface display plasmid pScaf-ctf were precultured in SDC medium (20 g/L dextrose, 5 g/L Bacto Casamino Acids, 6.7 g/L yeast nitrogen base without amino acids) at 30°C for 18h. For surface display of the scaffoldin, the precultured cells were inoculated into SGC medium (20 g/L galactose, 5 g/L Bacto Casamino Acids, 6.7 g/L yeast nitrogen base without amino acids) at OD_{600} of 0.5, and allowed to grow at 20°C for 60h. Wild-type *S. cerevisiae* EBY100 without the pScaf-ctf plasmid were cultured in Yeast Extract–Peptone–Dextrose (YPD) Medium (20 g/L dextrose, 20 g/L peptone, 10 g/L yeast extract).

Section S3: **Induction conditions for expression of PET-degrading enzymes**

E. coli SHuffleT7 was used as the expression host for dockerin-tagged FAST-PETase and MHETase. The cells harboring pBbE8k-FAST-PETase-Dockerin Rf/Cc were cultured overnight at 37°C in Lysogeny Broth (LB)-Miller medium (5 g/L yeast extract, 10 g/L NaCl, 10 g/L tryptone) containing kanamycin and scaled up at the same temperature until OD_{600} 0.9. Protein expression was induced with 10 mM L-Arabinose at 20°C/250RPM for 20h. The cells harboring pBbE8k-MHETase-Dockerin Ct were grown in Terrific Broth (TB) medium (24 g/L yeast extract, 20 g/L tryptone, 4 mL/L glycerol, 0.017 M KH_2PO_4, 0.072 M K_2HPO_4) and protein expression was induced with 1 mM L-Arabinose.

Section S4: HPLC method for analysis of enzymatic PET degradation products

The PET degradation products were analyzed using HPLC equipped with a diode array detector (Vanquish Core, ThermoFisher Scientific) using a ZORBAX Eclipse Plus C18 column (3.5 µm, 4.6 x 100 mm, Agilent). A linear gradient of 2 to 98%(v/v) acetonitrile (ACN) in water with 0.01% trifluoroacetic acid (TFA) over 15 min at a flow rate of 0.5 mL/min was used to separate the degradation products. The detection wavelength for the analytes was 240 nm. The following liquid chromatography conditions were used: 0 to 3 min, 2% ACN/0.01%TFA; 3 to 18 min, 2%-98%ACN/0.01%TFA (linear gradient); 18 to 21 min, 98% ACN/0.01%TFA; 21 to 26 min 2% ACN/0.01%TFA. The injection volume was 5µL.

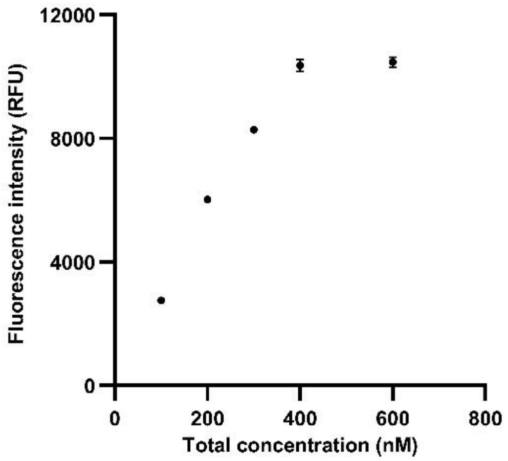

Figure S1: Determination of saturation concentration of FAST-PETase on the yeast scaffoldin (OD_{600} 5) after incubation with equimolar concentrations of FP-Cc and FP-Rf. The x-axis represents total FAST-PETase concentration. Fluorescence intensity was determined after incubation with anti-C-His antibody conjugated to Alexa Fluor 488. Experiments were conducted in triplicates, data shown are mean values (± standard deviation). Error bars smaller than size of symbol are not visible.

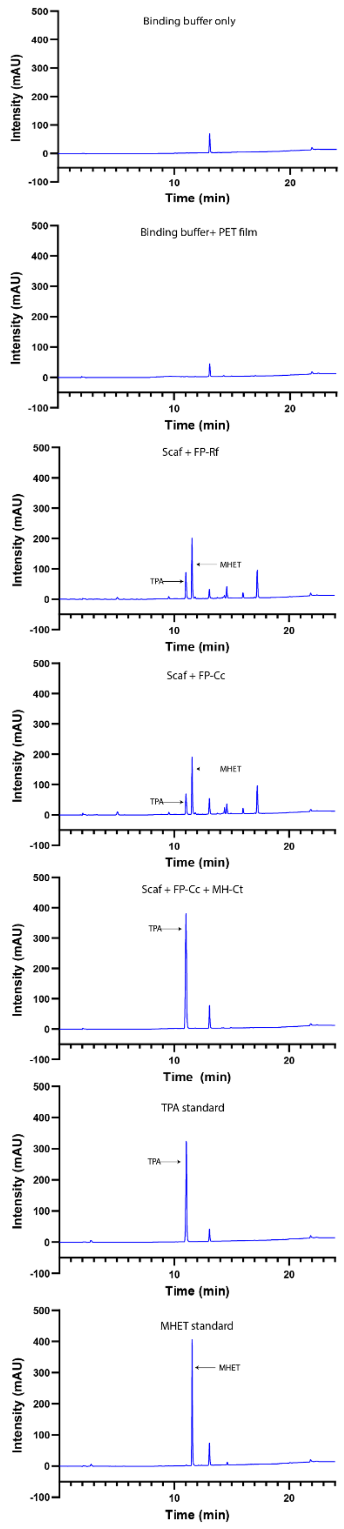

Figure S2: HPLC chromatograms of TPA and MHET standards, and PET degradation products on Day 4. All experiments were conducted using Binding buffer as reaction medium.

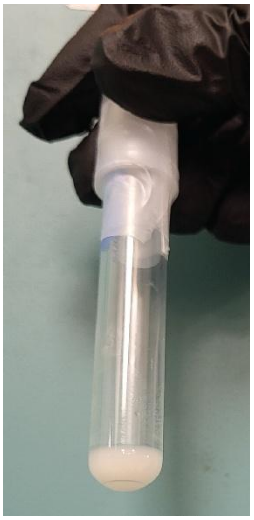

Figure S3: Reaction setup for PET depolymerization. One PET film (seen at the bottom of the glass test tube) was added to a test tube containing 300µL of yeast cells (OD_{600} 10).

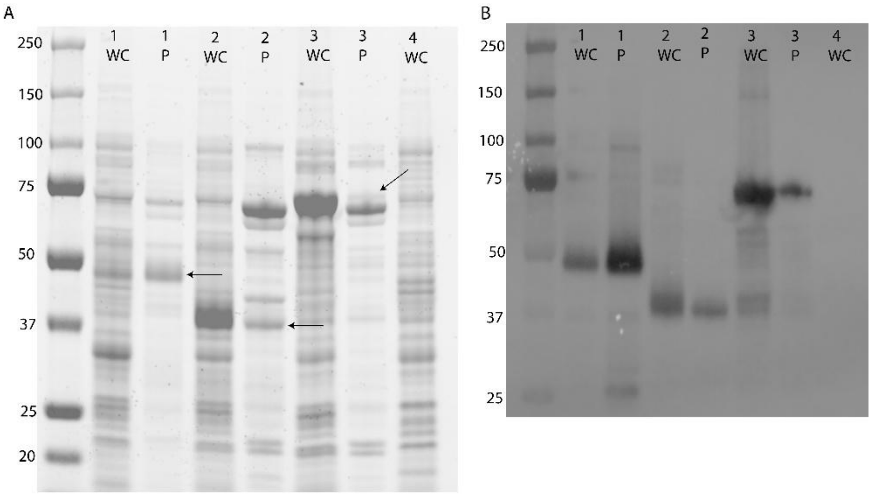

Sample number	Protein	Expected size (kDa)	Yield of protein of interest (mg protein/L culture)
1	FAST-PETase-Dockerin Rf	48.2	0.482
2	FAST-PETase-Dockerin Cc	43.0	0.390
3	MHETase-Dockerin Ct	74.4	0.203
4	Wild-type *E. coli* SHuffle T7	-	-
WC –*E. coli* Whole cell, P – Histag-purified protein			

Figure S4: (A) SDS-PAGE, **(B)** Western Blot [using anti-His$_6$ antibody] and yield of PET-degrading enzymes after Histag purification. 10% polyacrylamide SDS-PAGE gels (BioRad) were used for SDS-PAGE. For western blot, proteins were transferred to a polyvinylidene fluoride (PVDF) membrane, followed by blocking and incubation with a Horseradish peroxidase (HRP)-conjugated anti-His$_6$ antibody. Arrows in (A) indicate the band of interest.
